# Inter- and Intra-individual Variations in Foveal Outer Nuclear Layer Thickness and Their Associations with Clinical Characteristics in a Healthy Chinese Population

**DOI:** 10.1155/2020/7967393

**Published:** 2020-05-24

**Authors:** Jia Yu, Lei Li, Chunhui Jiang, Qing Chang, Qi Zhao

**Affiliations:** ^1^Department of Ophthalmology, Eye & Ear, Nose & Throat Hospital of Fudan University, Shanghai, China; ^2^Shanghai Key Laboratory of Visual Impairment and Restoration, Fudan University, Shanghai, China; ^3^NHC Key Laboratory of Myopia (Fudan University), Shanghai, China; ^4^Laboratory of Myopia, Chinese Academy of Medical Sciences, Shanghai 200031, China; ^5^Department of Epidemiology, School of Public Health, Fudan University, Shanghai 200032, China

## Abstract

**Purpose:**

To evaluate foveal outer nuclear layer (ONL) thickness and the difference thereof between bilateral eyes and their possible associations with clinical characteristics in a healthy Chinese population.

**Materials and Methods:**

Normal subjects were enrolled. Generalized linear models were used to assess the associations of foveal ONL thickness with sex, age, and spherical equivalents (SEs) and the associations of the difference in foveal ONL thickness between bilateral eyes with sex, age, and difference in SEs between bilateral eyes.

**Results:**

Totally, 304 subjects were included. The average foveal ONL thickness was 103.19 ± 14.25 (range 70–151) *μ*m in the right eye and 103.90 ± 14.63 (range 69–155) *μ*m in the left eye. The mean difference in foveal ONL thickness between right and left eyes was −0.71 ± 4.36 (range −13 to +12) *μ*m. Men had slightly greater foveal ONL thickness values in both right and left eyes compared with women (both *P* < 0.05); however, some women had a thicker foveal ONL than that of men (85/198 vs. 46/106 in the right eye; 79/198 vs. 52/106in the left eye). Age and SEs were not associated with foveal ONL thickness in either eye (all *P* > 0.05). Sex, age, and difference in SEs between bilateral eyes were not associated with the difference in foveal ONL thickness between bilateral eyes (all *P* > 0.05).

**Conclusions:**

Foveal ONL thickness showed wide variation in a normal Chinese population but little difference between bilateral eyes. Both these parameters could not be adjusted by sex, age, SEs, or the SEs difference between bilateral eyes. Thus, in those diseases involving only one eye, the difference or ratio of foveal ONL thickness between the affected eye and normal fellow eye may reflect the actual degree of the disease, rather than the foveal ONL thickness in the affected eye alone.

## 1. Introduction

The outer nuclear layer (ONL) of the retina contains the nuclei of cone and rod photoreceptors [1], and a reduction in ONL thickness is considered to be caused by photoreceptor cell death [2–6]. Thinning of the foveal ONL is associated with decreased visual acuity in various diseases; thus, foveal ONL thickness on optical coherence tomography (OCT) images is considered an important biomarker of retinal degeneration in clinical studies [7–14].

The great inter individual variability of the foveal ONL thickness among ethnicities has been reported [15]. However, the degree of variability in foveal ONL thickness in the normal Asian population and the associations of foveal ONL thickness with clinical characteristics remain unclear. Therefore, in this study, foveal ONL thickness and the difference thereof between bilateral eyes of the same subject and their possible associations with clinical characteristics were evaluated in a normal Chinese population.

## 2. Materials and Methods

The approval of the Ethics Committee of Eye and Ear Nose Throat (ENT) Hospital Fudan University was obtained, and written informed consent was obtained from all subjects before their enrolment in the study. The study adhered to the tenets of the Declaration of Helsinki.

### 2.1. Subjects

This was a prospective cross-sectional study that enrolled normal subjects of East Asian descent originating from mainland China, based on self-declaration, during routine ophthalmological examinations in the Eye, Ear, Nose, and Throat Hospital of Fudan University, Shanghai, China, from September 2014 to November 2019.

Data from both eyes were collected, including best corrected visual acuity (BCVA), measured using a standard Snellen chart and converted to logarithm of the minimum angle of resolution (logMAR) for statistical analysis; refraction data, converted to spherical equivalents (SEs), which were calculated as the spherical dioptric power plus one-half the cylindrical dioptric power; intraocular pressure (IOP), measured using a noncontact tonometer (TX-20, Canon, Tokyo, Japan); as well as the physical examination information of slit-lamp biomicroscopy and OCT examination.

The inclusion criteria of this study were SEs difference, defined as a difference in SEs between the right and left eyes of the same subject within −2.5 to +2.5 dioptres [D]; SEs in both eyes within −4 to +3 *D*; BCVA ≥ 20/25 in both eyes; no history of refractive surgery; IOP of 12–21 mmHg; no clinical signs or history of intraocular disease; and no staphyloma or choroidal excavation on any OCT image.

### 2.2. OCT Protocol

All OCT images were obtained through a dilated pupil using a high-definition 5-line raster scan protocol (length 6 mm, spacing 0.075 mm; Cirrus HD-OCT, Carl Zeiss Meditec, Dublin, CA, USA). In each subject, this protocol was applied horizontally and centred on the fovea. The OCT images that passed through the central fovea showing the steepest foveal excavation and the thinnest highly reflective Henle fibre layer were selected for measurement of ONL thickness [12]. Considering the potential diurnal variations in retinal layer thickness [16], we acquired all OCT images between 1 : 00 pm and 5 : 00 pm and within an interval of 3 min between bilateral eyes.

### 2.3. OCT Image Analysis

**Figure 1:**
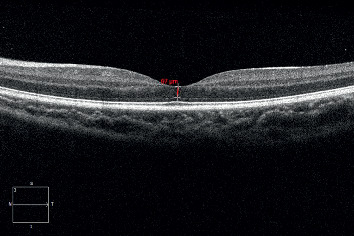
Illustration of foveal outer nuclear layer (ONL) thickness measurement in optical coherence tomography images. Foveal ONL thickness was defined as the distance between the internal limiting membrane and the external limiting membrane at the centre of the fovea.

Foveal ONL thickness was defined as the distances between the internal limiting membrane (ILM) and the external limiting membrane (ELM) at the centre of the fovea (Figure 1). The difference in the foveal ONL thickness was defined as the difference between the foveal ONL thickness measured from the right eye and that from the left eye in the same subject. The measurements were made manually using the supplied software (SW version 7.0.1.290, Carl Zeiss Meditec).

All scans and measurements were made by Y.J. The repeatability of the measurements was calculated from two horizontal scans taken in each eye during a single visit; 22 normal eyes were included to assess the repeatability of the measurements. Both the paired *t*-test and intraclass correlation coefficients (ICCs) were used to assess the repeatability of the measurements (an ICC of 0.81–1.00 indicated almost perfect agreement between repeated measurements, and an ICC < 0.40 indicated poor to fair agreement) [17].

### 2.4. Statistical Analysis

The data were analysed using SPSS for Windows version 21.0 (SPSS, Chicago, IL, USA). The calculated values are presented as either medians (P25 and P75), means ± standard deviations (SDs), or frequencies (proportions). The Kolmogorov–Smirnov test was used to confirm the normality of the data. The generalized linear models were used to assess the associations of foveal ONL thickness (dependent variable of interest) with sex, age, and SEs (independent variables), and the associations of the difference in foveal ONL thickness between bilateral eyes (dependent variable of interest) with sex, age, and the difference in SEs between bilateral eyes (independent variables). The paired *t*-test was used to assess the difference in foveal ONL thickness between bilateral eyes. Either Pearson correlation coefficient or Spearman correlation coefficient was used to examine the correlation between foveal ONL thickness of the bilateral eyes. A *P* value < 0.05 was considered statistically significant.

## 3. Results

**Figure 2:**
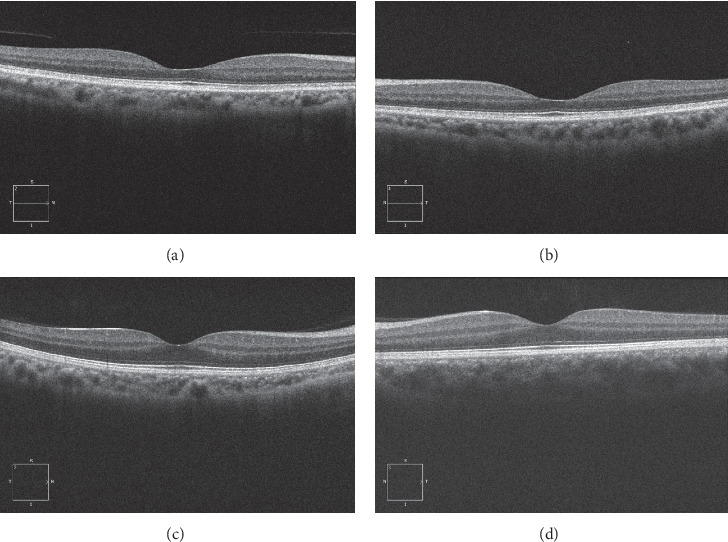
Representative images showing variation in the thickness of the foveal outer nuclear layer (ONL) among individuals in a normal Chinese population. (a, b) Horizontal line scans of the right and left eyes of a 61-year-old female subject with best corrected visual acuity (BCVA) of 20/20 in both eyes. The spherical equivalents (SEs) in the right and left eyes were +1 dioptres D and +1.25 D, respectively. The foveal ONL thicknesses in the right and left eyes were 75 and 70 *μ*m, respectively. (c, d) Horizontal line scans of the right and left eyes of a 29-year-old male subject with BCVA of 20/20 in both eyes. The SEs in the right and left eyes were −2 D and −2.5 D, respectively. The foveal ONL thicknesses in the right and left eyes were both 134 *μ*m.

A total of 304 subjects (608 eyes) were enrolled in the study. The demographic data for the subjects and the values for their clinical characteristics are listed in Table 1. The foveal ONL thickness varied greatly among *individuals*, while the difference in foveal ONL thickness between bilateral eyes was small (Table 1, Figure 2). The foveal ONL thickness measurements in normal eyes showed good repeatability (*t* = 1.183, *P*=0.250, paired *t*-test), with an ICC of 0.990.

Table 2 shows the regression coefficients (*β*) obtained from linear regression analyses of the associations of sex, age, and SEs (exposure variables of interest) with foveal ONL thickness (dependent variable).Men had slightly greater foveal ONL thickness values in both the right and left eyes compared with women (Table 2, both *P* < 0.05). However, some women had a thicker foveal ONL than that of men in either the right or left eye. In the right eye, 85 (42.9%) women had a foveal ONL thickness greater than the mean value (103.19), whereas 46 (43.4%) men had a foveal ONL thickness less than 103.19; in the left eye, 79 (39.9%) women had a foveal ONL thickness greater than the mean value (103.90), whereas 52 (49.1%) men had a foveal ONL thickness less than 103.90. Age and SEs were not associated with foveal ONL thickness in either the right or left eye (Table 2, both *P* > 0.05).

Table 2 also shows the regression coefficients determined by linear regression analyses of the associations of sex, age, and difference in SEs between bilateral eyes (exposure variables of interest) with the difference in foveal ONL thickness between bilateral eyes (dependent variable). No association of sex, age, or SEs difference between bilateral eyes with the difference in foveal ONL thickness between bilateral eyes was found (*P*=0.755, 0.513, or 0.653, respectively). The foveal ONL thickness was statistically different between bilateral eyes of the same subject (*t* = −2.831, *P*=0.005, paired *t*-test). However, the mean difference in the foveal ONL thickness of the right eye and the left eye was only −0.71 ± 4.36 *μ*m, and the foveal ONL thickness values of the bilateral eyes of the same subject were closely correlated (*r* = 0.955, *P*=0.0001).

## 4. Discussion

The study showed that in a healthy Chinese population, the foveal ONL thickness varied greatly, from 69 to 155 *μ*m with a SD of 15 *μ*m, while the difference in foveal ONL thickness between bilateral eyes was small (0.71 *μ*m), ranging from −13 to +12 *μ*m with a SD of 4 *μ*m. The foveal ONL thickness and the difference thereof between bilateral eyes were not associated or only weakly associated with sex, age, SEs, or the SEs difference between bilateral eyes.

Pilat et al. reported a notable difference in foveal ONL thickness among ethnicities [15]. In the present study, although strictly controlled healthy Chinese subjects were enrolled, the foveal ONL thickness varied greatly with the maximum value more than double the minimum value (155 vs. 69 *μ*m, respectively).This was probably related to the significant variation in foveal pit morphology among individuals [18–20]. Scheibe et al. evaluated the shape of the fovea in normal Caucasian subjects of European descent using OCT parameters including the foveal radius, foveal bowl area, and foveal rim height, all of which showed large differences among individuals [20]. This may partially explain the significant variation in foveal ONL thickness among the normal Chinese subjects in the present study. In contrast to the high variation among subjects, the foveal ONL thickness showed minimal differences between bilateral eyes of the same subject. This may be because the foveal region in bilateral eyes has good symmetry: foveal radius, foveal bowl area, foveal pit depth, and maximum slope, all of which exhibited high correlations between the right and left eyes of the same subject [18, 20].

It has been reported that the foveal cone density is highly variable among individuals, whereas the foveal cone densities of bilateral eyes are very similar [21–25].These findings may also partially explain the inter- and intra-individual variability in foveal ONL thickness in the present study.

Our study showed that foveal ONL thickness was associated with sex but not with age or SEs (Table 2). No previous study has examined these correlations using manual measurements of foveal ONL thickness. However, it has been reported that the minimum central retinal thickness (the distance between the ILM and retinal pigment epithelium at the foveal dip) is greater in men than women [26]. This finding was comparable to that of the current study. Although sex was associated with foveal ONL thickness, some women had a thicker foveal ONL than that of men (85/198 vs. 46/106 in the right eye; and 79/198 vs. 52/106 in the left eye). This finding suggested that only adjusting for sex in the comparison of foveal ONL thickness among individuals may be insufficient.

In addition, the ONL thickness within 1 mm region in early treatment diabetic retinopathy study circles by automated segmentations was not related to either age or SEs [27–30].These findings are similar to those of the current study. The present findings revealed a large variation in foveal ONL thickness in a normal Chinese population, and this variation was not associated with age or SEs. It suggested that the foveal ONL thickness cannot be adjusted by age or SEs when compared among individuals. Until the predictors of foveal ONL thickness are identified, directly comparing foveal ONL thickness values in the affected eyes among individuals could introduce bias or errors, especially in those clinical studies designed to detect only a very small degree of change in foveal ONL thickness.

In contrast, the difference in foveal ONL thickness between bilateral eyes was minimal (−0.71 ± 4.36 *μ*m) and was not associated with sex, age, or SEs difference. Although the foveal ONL thickness values of the bilateral eyes were statistically different (*t* = −2.831, *P*=0.005, paired *t*-test), they were closely correlated (*r* = 0.955, *P*=0.0001). This indicated that the foveal ONL thickness values in bilateral eyes of the same subject were almost the same, regardless of sex, age, or small SEs differences between bilateral eyes. Thus, in diseases involving only one eye, such as retinal detachment, and central serous chorioretinopathy (CSC), the normal fellow eye could serve as a reliable control; the difference or ratio of foveal ONL thickness between the affected eye and normal fellow eye may reflect the actual degree of the disease, rather than the foveal ONL thickness in the affected eye alone.

The normal ONL contains rod and cone photoreceptor nuclei throughout the retina with the exception of a rod-free zone approximately 350 *μ*m in diameter, extending 100 to 200 *μ*m from the foveal centre, in which only cones are present [21, 22]. A reduction in ONL thickness is considered to be caused by photoreceptor cell death [2–6]. Therefore, the ONL thickness can provide an indirect measure for the photoreceptor survival both to study the natural cause of degenerative retinal diseases and therapeutic effects of potential treatments. Henle fibre layer (HFL) consists of the photoreceptor axons, and Müller cell processes that are substantial in the human macula [31]. Several studies have reported that the HFL is routinely included in manual and automated segmentations of the apparent ONL, thus resulting in an artificially thick assessment of the true ONL thickness in spectral domain OCT images [32–38]. However, at the centre of the fovea, the Henle fibre layer is the thinnest, comprising less than 10.7% of the measured thickness [32]. Compared with using directional OCT followed by analyses in custom software [32], which can provide accurate ONL measurements, manual measurement of the ONL thickness at the centre of the fovea seems to be a simple and relatively accurate method in studies using spectral domain OCT, especially under pathological conditions such as retinal detachment and CSC.

Our study has several limitations: (1) axial length was not measured. As the range in SEs was small and the BCVA values were normal, the axial length may not have significantly affected the results; (2) only eyes with low refractive errors were included; (3) visual function tests were incomplete, including visual field test and colour vision test; and (4) the sample size was relatively small. Further studies that include axial length, complete visual function tests, and a larger sample size with greater refractive errors could provide more information.

## 5. Conclusion

In a normal Chinese population, the foveal ONL thickness varied greatly, while the difference in foveal ONL thickness between bilateral eyes was small. These two values could not be adjusted by sex, age, SEs, or the SEs difference between bilateral eyes. Thus, in those diseases involving only one eye, the difference or ratio of foveal ONL thickness between the affected eye and normal fellow eye may reflect the actual degree of the disease, rather than the foveal ONL thickness value of the affected eye alone.

## Figures and Tables

**Table 1:** The demographic data for the subjects and the values for their clinical characteristics.

	Mean	SD	Median	Min	Max	*P* ^*∗*^
Age (years)	50.43	16.06	**53.00**	20	80	0.0001
Male, *n* (%)	106 (34.9%)	NA	NA	NA	NA	NA
BCVA (logMAR) of the right eye	0.02	0.04	**0.00**	0.00	0.10	0.0001
BCVA (logMAR) of the left eye	0.02	0.04	**0.00**	0.00	0.10	0.0001
SEs of the right eye	−0.25	1.35	**0.00**	−3.75	2.75	0.0001
SEs of the left eye	−0.14	1.29	**0.13**	−3.88	2.88	0.0001
SEs difference	−0.12	0.63	−**0.13**	−2.38	1.88	0.0001
Foveal ONL thickness in the right eyes (*μ*m)	**103.19**	14.25	101.50	70	151	0.177
Foveal ONL thickness in the left eyes (*μ*m)	**103.90**	14.63	102.00	69	155	0.050
Difference in foveal ONL thickness	−**0.71**	4.36	0.00	−13	12	0.093

BCVA = best corrected visual acuity; N*A* = not applicable; logMAR = logarithm of the minimum angle of resolution; ONL = outer nuclear layer; SEs = spherical equivalents; SD = standard deviation; SEs difference was defined as a difference in SEs between the right and left eyes of the same subject. Difference in foveal ONL thickness was defined as the difference between the foveal ONL thickness measured from the right eye and that from the left eye in the same subject. ^*∗*^The Kolmogorov–Smirnov test was used to confirm the normality of the data. *P* ≥ 0.05 indicates that the data are normally distributed, and the mean value is shown in bold. *P* < 0.05 indicates that the data are not normally distributed, and the median value is shown in bold.

**Table 2:** The associations of foveal outer nuclear layer thickness with clinical characteristics using generalized linear models.

		The foveal ONL thickness in the right eyes		The foveal ONL thickness in the left eyes		The difference in foveal ONL thickness between the right eye and the left eye of the same subject	
		Beta (95% CI)	*P* value	Beta (95% CI)	*P* value	Beta (95% CI)	*P* value
Sex (M : F)		4.343 (0.967, 7.719)	0.012^a^	4.520 (1.069, 7.970)	0.010^a^	−0.166(−1.208, 0.877)	0.755^a^
Age		0.064 (−0.039, 0.167)	0.225^a^	0.075 (−0.030, 0.181)	0.162^a^	−0.010 (−0.041, 0.021)	0.513^a^
SEs		−0.354 (−1.581, 0.873)	0.571^a^	−0.430 (−1.734, 0.875)	0.518^a^		
The difference in SEs between the right eye and the left eye of the same subject						−0.178 (−0.954, 0.597)	0.653^a^

*F* = female; *M* = male; ONL = outer nuclear layer; SEs = spherical equivalents. Note that men had slightly greater foveal ONL thickness values in both the right and left eyes compared with women (both *P* < 0.05), whereas age and SEs were not associated with foveal ONL thickness in either the right or left eye (all *P* > 0.05). No association of sex, age, or SEs difference between bilateral eyes with the difference in foveal ONL thickness between bilateral eyes was found (*P*=0.755, 0.513, or 0.653, respectively) a generalized linear models.

## Data Availability

The data used to support the findings of this study are restricted by the Ethics Committee of Eye and Ear Nose Throat Hospital Fudan University in order to protect patient privacy. Data are available from Qing Chang (qngchang@aliyun.com) for researchers who meet the criteria for access to confidential data.

## Abbreviations

BCVA: Best corrected visual acuity
CSC: Central serous chorioretinopathy
ELM: External limiting membrane
ICCs: Intraclass correlation coefficients
ILM: Internal limiting membrane
IOP: Intraocular pressure
logMAR: Logarithm of the minimum angle of resolution
OCT: Optical coherence tomography
ONL: Outer nuclear layer
SDs: Standard deviations
SEs: Spherical equivalents.
